# Mysterious Civilizations: Is There a Connection between Medicine and Architecture in Ancient Egypt and Peru?

**DOI:** 10.7759/cureus.4576

**Published:** 2019-04-30

**Authors:** Karim ReFaey, William Clifton, Gabriella C Quinones, Shashwat Tripathi, Alfredo Quiñones-Hinojosa

**Affiliations:** 1 Neurosurgery, Mayo Clinic, Jacksonville, USA; 2 Art, University of Miami, Miami, USA; 3 Neurosurgery, University of Texas at Austin, Austin, USA

**Keywords:** ancient egyptian neurosurgery, ancient egyptian pyramids, ancient peruvian neurosurgery, ancient peruvian pyramids, great pyramids of giza, the great city of caral, history of neurosurgery

## Abstract

The ancient Egyptian and Peruvian Civilizations are two of the earliest cultures in human history. Through medical and architectural similarities, we wish to show a possible connection between these two cultures. A literature search was conducted by searching the database of Medline, National Geographic magazine, history books, and Google Scholar using the search terms: neurosurgery, pyramids, pyramidal architectures, ancient Egypt, ancient Peru, the connection between ancient Egypt and Peru. Both the ancient Egyptian and Peruvian Civilizations are well-known for their pioneering work in medicine and architecture; their accomplishments in these areas have been well-documented in the walls of uncovered tombs and discovered papyrus. Both these cultures also firmly believed in an afterlife and built pyramids to serve as tombs and homes for royalty in the afterlife. The sloping sides of the pyramids were inclined to signify the emergence of the physical body from Earth towards the Sun. Both civilizations independently pioneered the art of neurosurgery with different techniques and approaches. In this paper, we discuss the potential links between both civilizations. We recognize and appreciate the brilliance of these ancient cultures in mastering the medicinal and architectural sciences.

## Introduction and background

Neurosurgery as we know it flourished in the early twentieth century [1]. However, its roots date back to the golden ages of the ancient Egyptian and Latin American Civilizations [2-10]. The ancient Egyptians are well-known for pioneering medicine, highlighted by the excerebration and neurosurgical procedures conducted, as well as their architecture, as highlighted by their pyramidal architecture [2-4, 11-16]. The ancient Latin American culture, in particular, the Peruvians, performed skull trepanation and constructed similar pyramidal structures [5-10, 17-19]. Skull trepanation is a surgical intervention during which a hole is drilled into the skull exposing the underlying dura mater. This procedure was used to release pressure/blood or treat intracranial diseases. 

Despite the vast distance between the two civilizations, which were approximately more than 7,000 miles apart without known means of transport, surgical techniques and architectural similarities between the two ancient cultures were observed. Both, the ancient Egyptians and ancient Peruvians, had a strong religious belief in the afterlife; pyramids were built to honor their dead and act as a home in the afterlife. Both civilizations also had an astonishing connection within medicine: the ancient Egyptians and Peruvians mastered the integration of their medicinal knowledge and architecture to serve their religious beliefs of the afterlife. Both civilizations pioneered the art of neurosurgery with different techniques and approaches.

 We conducted a broad literature review by searching the database of Medline, National Geographic magazine, history books, and Google Scholar using the search terms: neurosurgery, pyramids, pyramidal architectures, ancient Egypt, ancient Peru, the connection between ancient Egypt and Peru. We could not find any original articles or attempts revealing the neurosurgical and architectural connection between the ancient Egyptian and Peruvian Civilizations. In this paper, we will discuss the potential hidden connection between both civilizations. Despite the lack of modern technologies and communication, both civilizations independently made similar advances in medicine and neurosurgery. We aim to acknowledge the intellect and the role of these ancient cultures in shaping modern medicine.

## Review

Egypt

The Nile Valley, in modern-day Egypt, was settled in approximately 6,000 BC by a Neolithic civilization followed by several independent cultures [20-21]. The first unified kingdom was led by King Menes in approximately 3150 BC; under the reign of King Menes, the culture thrived and made advancements in architecture, arts, and medicine. This dynasty was succeeded by the Old Kingdom (2700-2200 BC), during which many pyramids were built. Egypt has a rich history in the humanities and sciences and its influence can be seen throughout the ancient and modern worlds.

The brilliance of the ancient Egyptian Civilization can be seen in preserved writings and in paintings on tombs and temple walls. For example, inside the Egyptian tomb of Bani Hassan, a painting was found depicting a neurosurgical procedure in a sitting position [14]. The Edwin Smith papyrus and the Ebers papyrus, two of the oldest medical records, were both written in 1500 BC. The Edwin Smith papyrus describes a series of cases in the format of clinical examination, diagnosis, treatment, and prognosis. The central nervous system (CNS) section of the Edwin Smith papyrus contains 27 cases dedicated to head trauma, and six cases were dedicated to spinal trauma [2-3]. The head trauma cases were divided into three cases with head injuries, four cases with deep wounds in the scalp exposing the bone, and 11 cases with skull fractures [4]. The Ebers papyrus, on the other hand, describes different medical treatments for ailments such as migraines [22] and other medical conditions [23-24]. In addition to the Edwin Smith papyrus and Ebers papyrus, ancient Egyptians made paintings on the tombs and temple walls that described what we now know as hydrocephalus, hemiplegia, epistaxis with skull base fractures, and spine fractures. Furthermore, recent archeological findings suggest the presence of lesions resembling meningiomas in the skull of two different mummies [14].

**Figure 1:**
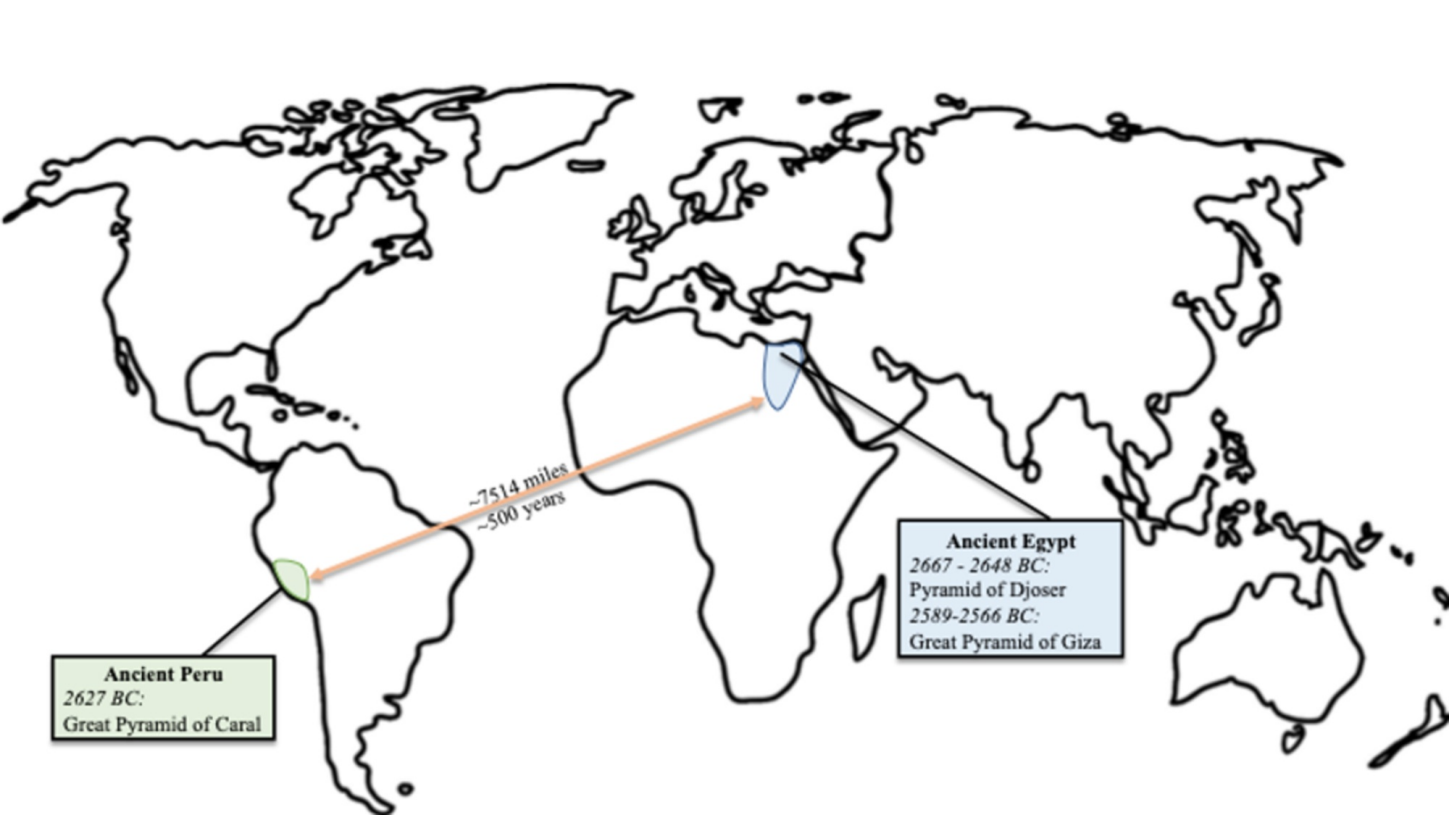
A world map highlighting the ancient Egyptian pyramids, dates, and location in blue; and the ancient Peruvian pyramids, dates, and locations in green; in addition to the distance and date differences between the two civilizations above and below the orange arrow, respectively

The ancient Egyptians realized that the removal of internal organs would help in the prevention of postmortem putrefaction, which afterward gave rise to the mummification process (late fourth millennium BC). The ancient Egyptians had a strong belief in the afterlife, and they believed that the soul had to be able to recognize the body to move onto the afterlife. Therefore, the prevention of postmortem putrefaction was imperative in ancient Egyptian culture [25]. During mummification rituals, the transnasal excerebration of the brain was performed. Transnasal excerebration is a procedure in which the brain is removed through the nose [11,15], which was adapted into the modern day trans-ethmoidal minimally invasive anterior skull base approach. Sources suggest that the minimally invasive techniques used in transnasal excerebration were developed for the use in trepanations [11,26]. Several trepanned skulls were found at excavation sites which were traced back to the ancient Egyptian Civilization between 2000 and 1788 BC (Figure 1) [11,26,27]. Reports show that trepanation and other surgical procedures were done under local anesthetic only, in which the combination of ground marble and vinegar was used to formulate anesthetic agents [14]. Ancient Egyptian surgeons had a variety of surgical tools and instruments including sutures, needles, scalpels, and materials for cauterization and hemostasis, where chisel and hammer were considered as the most used instruments for trepanation [11, 28]. Of note, archeologists suggest that the ancient Egyptians were so advanced in medical knowledge that most patients were treated conservatively using a nonsurgical approach, and surgical intervention was only used as a last resort due to the associated risks (Table 1) [28]. 

**Table 1:** A comparison between pyramids, neurosurgery, and religion in ancient Egypt and Peru

	Peru	Egypt
Architecture	Dates back to the third millennium BC. Wide base and sloping towards the top. Tombs for royalty. Mummies were not embalmed, and organs left alone.	Dated back to the third millennium BC. Wide base and sloping towards the top. Tombs for royalty. The mummification process involved the removal of internal organs to prevent postmortem putrefaction.
Neurosurgery	No known document. Quadrilateral trepanation with burr holes, consisting of four rectilinear incisions. First verified case of trepanation for a therapeutic procedure. Performed trepanation on all areas of the skull. Considered “center of trepanations”. Left-sided procedures. Performed more trepanations than the Egyptians.	Wrote the Edwin papyrus. Paintings on walls of tombs showing hydrocephalus, hemiplegia, epistaxis with skull-base fractures, and spine fractures. Transnasal excerebration (foundation for the modern trans-ethmoidal minimally invasive anterior skull base approach). Documented use of local anesthetic. Left-sided procedures.
Religion	Stressed the importance of the afterlife. Worshipped the Sun God, Inti.	Stressed the importance of the afterlife. Worshipped the Sun God, Ka.

Once the mummification rituals were completed using the previously mentioned tools and techniques, the mummies were honored in their final shrines. All of the belongings and fortunes of the deceased were placed near the sarcophagus, and the preparation for life after death culminated in the pyramids. The mummies’ arms were crossed, to signify the balance of life and death. The ancient Egyptians believed that a second entity existed in every human, called Ka. When the physical body terminated, the Ka lived for eternity [29]. They did not see the death of the physical body as an ultimate end, but rather as a transitional phase. The sloping sides of the pyramids were inclined to signify the emerging of the physical body from the Earth towards the Sun. Some pyramids were even named after the sunshine. The ancient Egyptians pyramids (dated back to third millennium BC) were constructed to serve as tombs for kings and queens (Figure 1). The ancient Egyptian pyramids were astronomically oriented to the cardinal points with astonishing precision [12]. Limestone was the primary material used for the construction, giving the pyramid its shiny aspect, reflecting their belief in the sun as an essential entity. It is believed that limestone blocks were man-made, carved, and crafted to look like natural stones [13]. Ancient Egyptian paintings illustrate that the stone blocks were transported on sledges and moved over a lubricated surface, and then moved onto ramps for its final position. The exterior was then completed starting from the top to bottom, with the ramps removed after the work was completed (Table 1). 

Peru

Located on the other end of the world, the ancient Peruvians were astonishingly ahead of their time as well (Figure 1).

The Norte Chico or Caral-Supe Civilization (3500 BC) was located in the coastal region of modern day northern Peru and is often cited as the earliest settlement in Americas and lasted until approximately 1800 BC [30-31]. Among the civilization’s most prominent achievements were their impressive architecture, textiles, and medicine.

The ancient Peruvian Civilization was known for using the trepanation techniques as well, with the first verified case of trepanation on a living person for a therapeutic neurosurgical procedure being performed around 400 BC in the southern coast of Peru [5-10]. The ancient Peruvians developed a slightly different trepanation technique than that of the Egyptians, which is known as quadrilateral trepanation; the burr holes consisted of four rectilinear incisions [9-10]. This technique subsequently spread to various areas in the Peruvian highlands and was in practice until the Incan conquest in 1532 AD [7-8]. Many of the trepanned skulls demonstrated evidence of survival, but no biological activity. Therefore, it has been concluded that these procedures were performed immediately before or after death [8-9]. Josiah C. Nott was a physician who made attempts to reveal the mystery behind the trepanation procedure. Unlike the ancient Egyptians, ancient Peruvians performed trepanation on all areas of the skull except for the skull base or regions covered entirely by muscle (Table 1) [19]. While Nott believed that the trepanations were performed to treat skull trauma caused during fighting, Paul Broca, an iconic neuroscientist and neurosurgeon, hypothesized that trepanation was used to treat convulsions in infants [19,32]. In the end, many scientists have concluded that the trepanations were performed to drain epidural hematomas due to the absence of any fractures, fissures, or other types of trauma [9-10]. Trepanation was used in both of these societies for medical and religious purposes. Interestingly, both these cultures preferred left-sided procedures, likely due to the anatomic location of the heart [33]. Based on current evidence, it appears that Peruvians performed more trepanations and subsequently made more discoveries in trepanation than the Egyptians. Peru, therefore, is considered the center of trepanations [34-36].

Similar to the ancient Egyptians, ancient Peruvians placed great importance on the afterlife, and this impacted their daily life, architectural structures, and medicine. The spiritual aspect was based upon the sun. The solar divinity was Inti [17]. Peruvians used to build pyramids to serve as tombs for their royalty and a home in the afterlife, which resembles the ancient Egyptian pyramids in the aspect of the wide base and sloping walls towards the top. The stones were meticulously crafted, leaving no room for errors. However, the Peruvians did not use the same mummification and burial techniques as the Egyptians [37]. Peruvian mummies were not embalmed and their organs were left inside [37].

## Conclusions

Ancient Egyptians and Peruvians were leaders in medicine and architecture. This is clearly documented in ancient writings and on the walls of tombs and temples in Egypt and Peru. As pioneer healers and master architects who were driven by a sincere religious belief in death and the afterlife, ancient Egyptians and Peruvians devised their medicinal and architectural knowledge to serve their faith and beliefs. This reveals the progressive nature of these ancient cultures considering the unavailability of any radiographic or computational technologies. The significance of our hypothesis is the recognition and appreciation of the virtuosity and sagacity of the ancient cultures in mastering the medicinal and architectural sciences.
